# Colonization of the Intestinal Tract of the Polyphagous Pest *Spodoptera littoralis* with the GFP-Tagged Indigenous Gut Bacterium *Enterococcus mundtii*

**DOI:** 10.3389/fmicb.2016.00928

**Published:** 2016-06-14

**Authors:** Beng-Soon Teh, Johanna Apel, Yongqi Shao, Wilhelm Boland

**Affiliations:** ^1^Department of Bioorganic Chemistry, Max Planck Institute for Chemical EcologyJena, Germany; ^2^Clinic for Internal Medicine II, Department of Haematology and Medical OncologyUniversity Hospital Jena, Germany; ^3^Laboratory of Invertebrate Pathology, College of Animal Sciences, Zhejiang UniversityHangzhou, China

**Keywords:** *Spodoptera littoralis*, green fluorescent protein, promoter, lactic acid bacteria, *Enterococcus mundtii*, intestinal tract

## Abstract

The alkaline gut of Lepidopterans plays a crucial role in shaping communities of bacteria. *Enterococcus mundtii* has emerged as one of the predominant gut microorganisms in the gastrointestinal tract of the major agricultural pest, *Spodoptera littoralis*. Therefore, it was selected as a model bacterium to study its adaptation to harsh alkaline gut conditions in its host insect throughout different stages of development (larvae, pupae, adults, and eggs). To date, the mechanism of bacterial survival in insects' intestinal tract has been unknown. Therefore, we have engineered a GFP-tagged species of bacteria, *E. mundtii*, to track how it colonizes the intestine of *S. littoralis*. Three promoters of different strengths were used to control the expression of GFP in *E. mundtii*. The promoter *ermB* was the most effective, exhibiting the highest GFP fluorescence intensity, and hence was chosen as our main construct. Our data show that the engineered fluorescent bacteria survived and proliferated in the intestinal tract of the insect at all life stages for up to the second generation following ingestion.

## Introduction

Insects' guts harbor a wide range of microbial communities. Intestinal gut microbes contribute significantly to the development of their insect hosts by providing essential nutrients, aiding in food digestion, and protecting against other harmful pathogens. However, the functions of these microbes in the insect gut are still largely unknown due to the complexity and diversity of the microbes. In recent years, the agricultural pest, *Spodoptera littoralis* (Lepidoptera, Noctuidae) has been used as an experimental model insect to study gut microbiomes. The microbial composition in the gut of *S. littoralis* has been well characterized (Tang et al., 2012), yet the factors controlling its colonization are unknown.

Insect guts contain multiple compartments with different physicochemical conditions such as pH and oxygen availability which enrich for certain species of bacteria. The gut of certain lepidopteran, coleopteran, and dipteran is highly alkaline due to specific dietary preferences (Brune and Kühl, 1996; Harrison, 2001). The lepidopteran insects which feed on tannin-rich leaves have alkaline midguts with pH as high as 11–12 (Appel and Martin, 1990; Harrison, 2001). A clear pH gradient occurs along the lepidopteran midgut from highly alkaline (pH ~ 10) anterior end to almost neutral posterior ends (Funke et al., 2008). The microbial community of *S. littoralis* (cotton leafworm) is dominated by *Pantoea* and *Citrobacter* from the phylum Proteobacteria in early-instar larvae (Shao et al., 2014). Bacteria in this phylum have the ability to degrade polysaccharide in insects (Anand et al., 2010; Adams et al., 2011; Engel et al., 2012). As insects aged toward late-instar, more than 97% of the total bacterial community shifted to Firmicutes, dominated mostly by *Enterococcus* and *Clostridium* sp. (Tang et al., 2012; Shao et al., 2014). The proliferation of *Clostridia* is linked to its role in cellulose digestion and fermentation of sugars (Watanabe and Tokuda, 2010). Interestingly, the alkaline midgut of gypsy moth larva also harbors *Enterococcus* (Broderick et al., 2004) while the Firmicutes dominates the midgut of the beetle *Pachnoda ephippiata* (Egert et al., 2003). Some insects harbor similar bacterial lineages in their alkaline guts.

To date, the genus *Enterococcus* is known to include more than 33 species (Kohler, 2007). The members of this genus are typically found in the intestinal tracts of humans and animals, in dairy products, and also in the environment: for example, in plant material, soil, and surface water (Giraffa, 2003; Ogier and Serror, 2008). *E. mundtii* is part of this genus. It is a non-motile, Gram-positive, facultative anaerobic organism that belongs to the group of lactic acid bacteria (LAB). It forms either cocci or rods, and is capable of producing lactic acid as a by-product of the fermentation of carbohydrates. The biological role of *E. mundtii* is still poorly understood, as most studies have focused on the model bacteria *Enterococcus faecalis* and *Enterococcus faecium*, which often cause human systemic infection (Arias and Murray, 2012).

Green fluorescent protein (GFP) originally isolated from *Aequorea victoria* has been extensively used as a reporter for gene expression in bacterial and mammalian cells (Yang et al., 1996; Valdivia and Falkow, 1997; Hazelrigg et al., 1998; Rolls et al., 1999). GFP is advantageous as it requires neither cofactors nor a substrate to be expressed in its host cells. Different variants of GFP, such as EGFP (enhanced green fluorescent protein) have been developed to improve fluorescent intensity (Cormack et al., 1996). The expression of GFP in several LAB has been successfully demonstrated (Scott et al., 2000; Hansen et al., 2001; Lun and Willson, 2004). In recent years GFP has been mostly used to investigate Gram-negative bacteria, and, less often, Gram-positive bacteria (Bubert et al., 1999; Freitag and Jacobs, 1999; Lewis and Marston, 1999; Fernandez de Palencia et al., 2000). Increasingly, GFP has been used to track how and where target bacterial species colonize the guts of several host insects (Thimm et al., 1998; Mumcuoglu et al., 2001; Husseneder and Grace, 2005; Kounatidis et al., 2009; McGaughey and Nayduch, 2009; Doud and Zurek, 2012).

In this work, we determine the fate of GFP-tagged *E. mundtii* within the digestive tract of *S. littoralis* when administered *in vivo*. In addition, we track the transmission route of the bacteria through all stages of the life cycle of *S. littoralis*. In fact, the incorporation of GFP-tagged *E. mundtii* provides a non-invasive monitoring of its survival in the insect gut, but still far from addressing the relationship between the insect and the bacterial symbiont. We are interested to further explore the underlying factors that drive this complex relationship by analyzing the bacterial and insect gut epithelial transcriptomes in future work. The transcriptome data will significantly expand our understanding of the functional roles of indigenous bacteria toward the development of the insect and other microbes. This can easily be done by identifying the insect- or microbe-derived compounds from the metabolic pathways resulted from the transcriptome data.

## Materials and methods

### Maintenance of egg and larvae

The eggs of *S. littoralis* were purchased from Syngenta Crop Protection Münchwilen AG (Münchwilen, Switzerland). Eggs were hatched at 14°C. Larvae were maintained at room temperature (24°C). Larvae were provided with sterile artificial diet made of white bean and essential nutrients without antibiotics and prepared based on Spiteller et al. (2000).

### Bacterial strains and growth conditions

Table 1 lists the bacterial strains and plasmids used in this study. *Escherichia coli* strain DH5α was used to maintain all GFP-containing plasmids. The plasmid pTRKH3-*erm*GFP (Addgene plasmid # 27169), pTRKH3-*slp*GFP (Addgene plasmid # 27168), and pTRKH3-*ldh*GFP (Addgene plasmid # 27167) were gifts from Michela Lizier. *E. mundtii* strain KD251 (isolated from the gut of *S. littoralis* at the Department of Bioorganic Chemistry, Max Planck Institute for Chemical Ecology) was used as the recipient of all plasmids (Shao unpublished). *E. coli* DH5α and *E. mundtii* were grown at 37°C with agitation (220 rpm) in Luria-Bertani (LB) and Todd-Hewitt Bouillon, THB (Roth, Karlsruhe, Germany) medium for both broth and agar, respectively. Antibiotics were used at the following concentrations: erythromycin, 50 μg ml^−1^ (for *E. coli*) or 5 μg ml^−1^ (for *E. mundtii*). All plasmids were extracted from *E. coli* using the GeneJet plasmid miniprep kit (Thermo Scientific, Vilnius, Lithuania). All strains were kept in glycerol stocks at −80°C for preservation and long-term storage.

**Table 1:** **Bacterial strains and plasmids used in this study**.

**Strain or plasmid**	**Relevant properties**	**Reference or source**
**STRAINS**		
*E. mundtii* KD251	Transformation host, isolated from the intestine of *S. littoralis*	Laboratory collection
*E. coli* DH5α	Transformed bacteria in stab cultures	Addgene
**PLASMIDS**		
pTRKH3	7.8 kB, *E. coli*-LAB shuttle vector, Em^r^, Tet^r^, pAMß1 origin, p15A origin	O'Sullivan and Klaenhammer, 1993
pTRKH3-*erm*GFP	Em^r^, pAMß1 origin, p15A origin, pTRKH3 derivative containing e*gfp* gene downstream of *ermB* promoter	Addgene plasmid 27169
pTRKH3-*ldh*GFP	Em^r^, pAMß1 origin, p15A origin, pTRKH3 derivative containing e*gfp* gene downstream of *ldhL* promoter	Addgene plasmid 27167
pTRKH3-*slp*GFP	Em^r^, pAMß1 origin, p15A origin, pTRKH3 derivative containing e*gfp* gene downstream of *slp* promoter	Addgene plasmid 27168

*Em^r^, erythromycin resistant; Tet^r^, tetracycline resistant*.

### Plasmids

All GFP expression vectors were derived from pTRKH3, a backbone shuttle vector for *E. coli* and various species of LAB, including *Streptococcus, Lactococcus, Enterococcus*, and *Lactobacillus* (O'Sullivan and Klaenhammer, 1993). The vector carries a gene for erythromycin resistance which is highly suitable for expression in *Enterococcus*. In addition, the vector possesses a modified GFP 5 (mGFP5) that is controlled by three constitutive promoters of different strengths. pTRKH3-*erm*GFP harbors EGFP that is controlled by a strong enterococcal erythromycin ribosomal methylase (*ermB*) promoter (Swinfield et al., 1990). The *Lactobacillus acidophilus* lactate dehydrogenase (*ldhL*) promoter (Kim et al., 1991) and surface layer protein (*slp*) promoter (Boot and Pouwels, 1996) constitutively control pTRKH3-*ldh*GFP and pTRKH3-*slp*GFP, respectively.

### Electroporation of *Enterococcus mundtii*

Electroporation was carried out based on the modified protocol of *E. coli* (Dower et al., 1988). A single colony of *E. mundtii* was grown at 37°C in THB broth on a rotary shaker (Certomat BS-1 Sartorius, Goettingen, Germany) with agitation (220 rpm). An overnight culture was diluted 1:1000 in 100 ml of THB medium before being harvested by centrifugation at 4000 × g for 10 min (Sigma 3K18, Sigma, Germany) at 4°C when growth reached the exponential phase (A_600 nm_ approximately 2.2). The cells were washed with 100 ml of ice-cold distilled water, centrifuged as above and washed again with 50 ml of ice-cold water before being centrifuged again. The cells were then washed with 20 ml of 10% glycerol, centrifuged and finally suspended in 2 ml of 10% glycerol. The suspension was divided into 50 μl aliquots and stored at -80°C. Prior to electroporation, the frozen cells were thawed on ice and mixed with plasmid for 15 min before being transferred into a chilled 0.2 cm gap cuvette. Electroporation was performed by a single pulse at 1.8 kV (*E* = 9 kV/cm), 600 Ω and 10 μF, with a pulse length of 3.6 ms in an electroporator 2510 (Eppendorf, Hamburg, Germany). The concentration of purified plasmids used during electroporation was between 0.15 and 0.2 μg. The pulsed cells were immediately suspended with 950 μl of THB broth and further incubated for 2 h at 37°C with agitation (220 rpm) before 100 μl was plated on THB agar containing 5 μg ml^−1^ of erythromycin. The plates were incubated at 37°C for 48 h. Bacterial transformants containing target plasmids were verified by PCR screening.

### Verification of bacterial identity by 16S rRNA sequencing

All bacterial transformants were checked for identity by PCR to prevent contamination. Total DNA was extracted from GFP-tagged bacteria of three different constructs from overnight culture by using a MasterPure Complete DNA and RNA purification kit (Epicentre, Madison, WI, USA) according to the manufacturer's protocol. The bacterial 16S rRNA genes were amplified using universal primers, 27f (5′- AGAGTTTGATCCTGGCTCAG-3′) and 1492r (5′-GGTTACCTTGTTACGACTT-3′). PCR was performed in a final volume of 50 μl using 10 μM of each primer, 10 mM concentration of deoxynucleoside triphosphates, 50 mM MgCl_2_, 1 U of *Taq* polymerase and buffer (Invitrogen, Carlsbad, CA, USA). Denaturation was performed at 95°C for 2 min, followed by 30 cycles of 95°C for 30 s, annealing at 54°C for 30 s, and 72°C at 1 min 30 s. The final extension was at 72°C for 7 min. PCR products were purified using the PureLink Quick Gel Extraction and PCR Purification Combo Kit (Invitrogen, Carlsbad, CA, USA). The purified PCR products were sent for Sanger sequencing. DNA sequences were assembled with DNA baser sequence assembly software (http://www.dnabaser.com). The assembled sequences were used for blast searches at the National Center for Biotechnology Information (http://www.ncbi.nlm.nih.gov).

### Feeding of *S. littoralis* larvae with GFP bacteria

A total of 50 first-instar larvae were fed artificial diet supplemented with antibiotics for 3 days in the following final concentration: ampicillin (5.75 μg ml^−1^) and erythromycin (9.6 μg ml^−1^). Each larva was fed small cubes (1 g) of artificial diet inoculated with *E. mundtii*-harboring pTRKH3-*erm*GFP for 1 day starting at day 6, followed by food without bacteria starting at day 7 until pupation. Control larvae were fed food without bacteria continuously. A single colony of bacteria was grown overnight in THB broth containing erythromycin (5 μg ml^−1^) and diluted 1:10 in the same broth before being fed. A total of 100 μl from the 1:10 dilution broth (A_600 nm_ approximately 0.65) containing ~4.7 × 10^7^ CFUs of GFP bacteria was applied to the food of the larvae. Every day, feces were removed to avoid re-inoculating the GFP bacteria.

### Quantification of bacteria from the intestinal tract

Larvae (*n* = 6 for each stage), adults or pupae (*n* = 3 for each stage), and a control (*n* = 1 for each stage) were killed by freezing at −20°C for 15 min. Each individual was surface sterilized in 70% ethanol and immediately rinsed in sterile distilled water. Guts were dissected in sterile 1 × PBS (137 mM NaCl, 2.7 mM KCl, 10 mM Na_2_HPO_4_.7H_2_0, and 2 mM KH_2_PO_4_ [pH 7.4]), with sterile forceps under a stereomicroscope (Stemi 2000-C, Zeiss, Jena, Germany). Larval guts were excised into three sections: foregut, midgut, and hindgut. Gut tissues were aseptically homogenized in 100 μl of PBS. A serial dilution of 10-fold was performed by transferring 100 μl of the homogenized sample into 900 μl sterile PBS, vortexing vigorously, and spread-plating 100 μl of each dilution onto THB agar supplemented with erythromycin (5 μg ml^−1^). All plates were incubated at 37°C for 48 h. Total bacterial cells were counted as colony forming units (CFUs) for each intestinal tract region. Erythromycin resistance was used as selection marker for picking bacterial colonies. In addition, to verify the presence of plasmid-containing GFP, PCR screening was performed.

### Tissue cross-sectioning

The fresh gut tissues were cut into sections (foregut, midgut, and hindgut) and frozen at −24°C in mounting medium for cryotomy (OCT compound, VWR, Leuven, Belgium) for 30 min. They were then cut with cryomicrotome (Microm Cryo-Star HM560 Cryostat, Walldorf, Germany) into 14–100 μm sections.

### Fluorescence microscopy

The cultures containing GFP-producing bacteria were harvested, and the pellets were suspended in 1 × PBS. Bacterial suspensions of 20 μl or slices of cross-sectioned tissue were mounted on microscope slides (Superfrost Plus, Thermo Scientific). Live cells were observed under an Axio Imager Z1 fluorescent microscope equipped with an AxioCam MRm camera (Zeiss, Jena, Germany). The GFP signal was detected using the filter set 10 (Cy2/GFP). All images were captured with a 63X magnification oil objective with an aperture of 1.4. The images were analyzed using the Axio Vision Rel 4.8 software (Zeiss, Jena, Germany). ImageJ, Fiji (Schindelin et al., 2012), an open-source software, was used to process all fluorescent images.

### DNA extraction and PCR amplification of *gfp* gene

Total DNA was extracted from larvae and pupae at all instars, and from adults, by using a DNA kit as mentioned above. The 735 bp of *gfp* gene was amplified using a set of primers consisting of GFP3fw (5′-TCGGAATTCATGAGTAAAGGAGAAGAA-3′) and GFP3rev (5′- TCAGGATCCTTATTTGTATAGTTCATCC-3′) (Lizier et al., 2010). An EcoRI and a BamHI site (underlined) were introduced for forward and reverse primers, respectively. The PCRs were performed in a final volume of 20 μl using 10 μM of each primer, 10 mM concentration of deoxynucleoside triphosphates, 50 mM MgCl_2_, 1 U of *Taq* polymerase and buffer (Invitrogen, CA, USA). The following PCR conditions were used: 3 min at 94°C, followed by 35 cycles of 45 s at 94°C, 30 s at 60°C, and 2 min at 72°C, and final extension of 10 min at 72°C.

### Western blot

Bacterial cells were harvested from exponentially growing cultures. The cells were suspended in TE buffer (10 mM Tris-HCl, pH 8.0; 1 mM EDTA) containing 20% sucrose, lysozyme (1 mg ml^−1^), RNase (1 μg ml^−1^), and DNase (1 μg ml^−1^) and further disrupted by repeating a freeze-thaw cycle. The protein extracts were subjected to sodium dodecyl sulfate polyacrylamide gel electrophoresis (SDS-PAGE) on a 4–12% gel (Laemmli, 1970). The proteins were transferred onto a PVDF transfer membrane, pore size 0.45 μm (Thermo Scientific, Schwerte) with an electroblotter (Trans-blot Turbo Transfer System, BIO-RAD, Munich, Germany). The blots were blocked with 5% non-fat skimmed milk (NFDM) in TBS-T (Tris-buffer saline with Tween-20) for 1 h at room temperature. The membrane was then incubated for 16 h at 4°C with the mouse primary antibody anti-GFP (Roche Applied Science, Rotkreuz, Switzerland) diluted 1:2000 in blocking buffer. After three washes in blocking buffer, the membrane was incubated for 1 h at room temperature with Anti-mouse lgG, HRP-linked Antibody (Cell Signaling Technology, Cambridge, UK) diluted 1:5000 in blocking buffer. The membrane was washed three times in blocking buffer followed by incubation with the chemiluminescent reagent for 1 min. In the dark room, the membrane was transferred onto a foil, and X-ray film (CL-XPosure Film, Thermo Scientific, Schwerte, Germany) was placed on top of it. The film was developed after different exposure times (3 s–10 min).

### Flow cytometry

Bacteria from overnight cultures were re-suspended and diluted 1:10,000 in 1 × PBS. Fluorescence was determined in a CyFlow Space (Sysmex Partec, Görlitz, Germany). The data were analyzed using the CyFlow Space Operating Software FloMax. A blue laser (488 nm) was used for GFP fluorescence detection.

### Statistical analysis

Bacterial plate counts between 30 and 300 colonies were included in the calculation. Samples with colonies above 300 may not be distinguishable from one another on a plate count, whereas those below 30 may not be representative of the sample (Madigan et al., 2009). The total number of fluorescent *E. mundtii* recovered from each intestinal tract (foregut, midgut, and hindgut) across different larval stages was analyzed using JMP® 12.1.0^1^. Counts were analyzed using a one-way ANOVA test (*P* < 0.05). To further understand the different survival rates of GFP-*E. mundtii* at different larval stages, we compared the means of the combined three gut parts (foregut, midgut, and hindgut) as well as the means of individual gut regions of each larva using the Tukey–Kramer test (*P* < 0.05).

## Results

### Comparison of different GFP constructs

**Figure 1:**
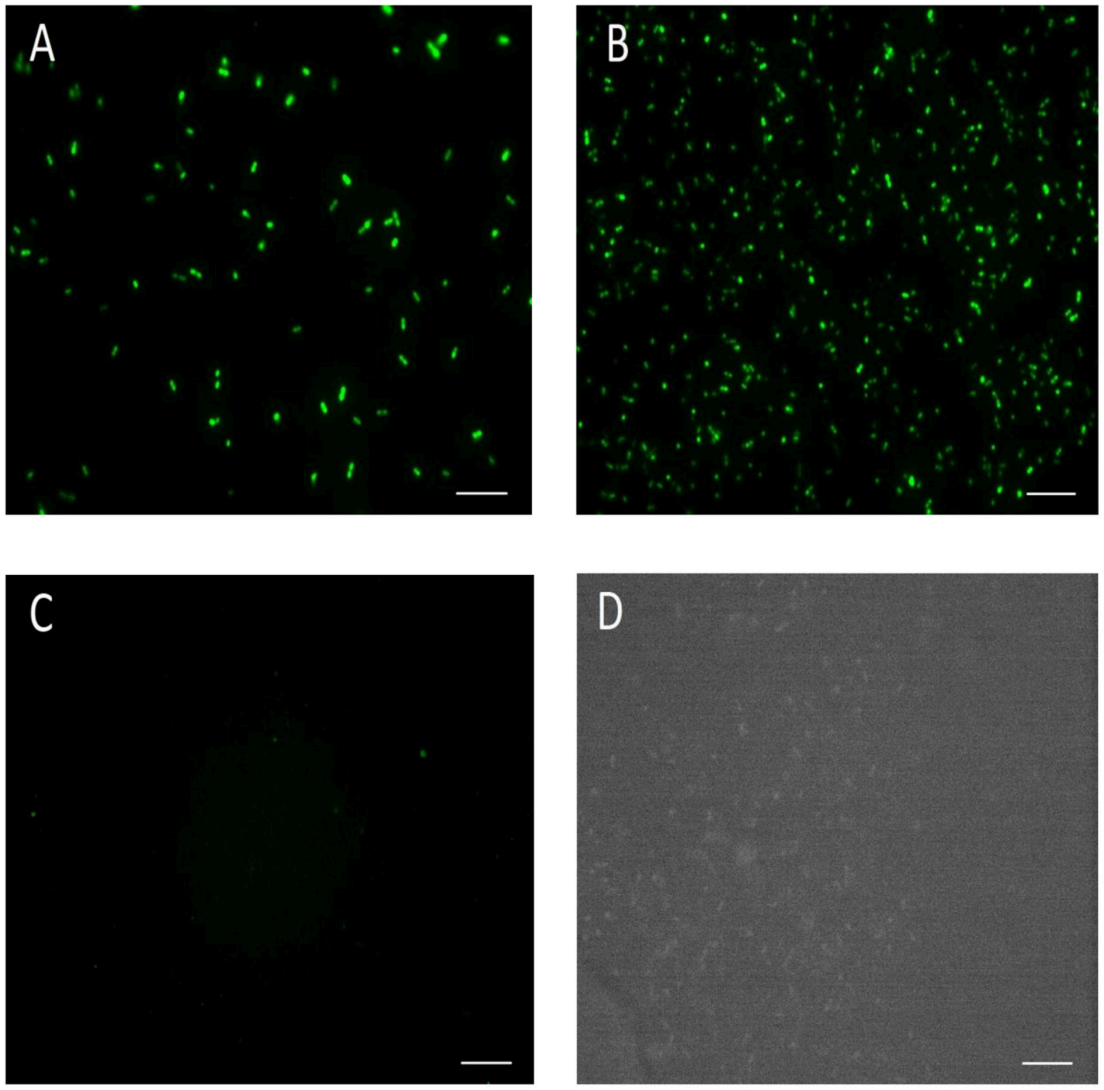
**Fluorescence micrographs of different constructs of GFP-expressing ***E. mundtii*** under the control of three constitutive promoters. (A)** Strain of *E. mundtii*/pTRKH3-*erm*GFP, **(B)** Strain of *E. mundtii*/pTRKH3-*ldh*GFP, **(C)** Strain of *E. mundtii*/pTRKH3-*slp*GFP, **(D)** *E. mundtii* wild type (control) grown in THB for 24 h. All recombinant bacterial strains were grown in THB with erythromycin for 24 h. Scale bars: 10 μm. Magnification, 630X.

Three different promoters, *ermB, ldhL*, and *slp* were used to control the expression of GFP, using pTRKH3 as a backbone shuttle vector. The strength of these GFP constructs was tested in *E. mundtii* by electroporation. This method was able to yield transformed colonies for all constructs. The recombinant bacterial colonies were picked and grown in THB at 37°C overnight before GFP fluorescence was visualized by epifluorescence microscopy. The highest fluorescence intensity was detected for *E. mundtii* transformed with pTRKH3-*erm*GFP (Figure 1A), followed by pTRKH3-*ldh*GFP (Figure 1B) and no signal for pTRKH3-*slp*GFP (Figure 1C) as well as wild-type *E. mundtii* (Figure 1D). The bacterial cultures were all grown simultaneously for 24 h, equivalent to stationary phase. The GFP content represents the same amount of cells which was measured as OD_600 nm_.

**Figure 2:**
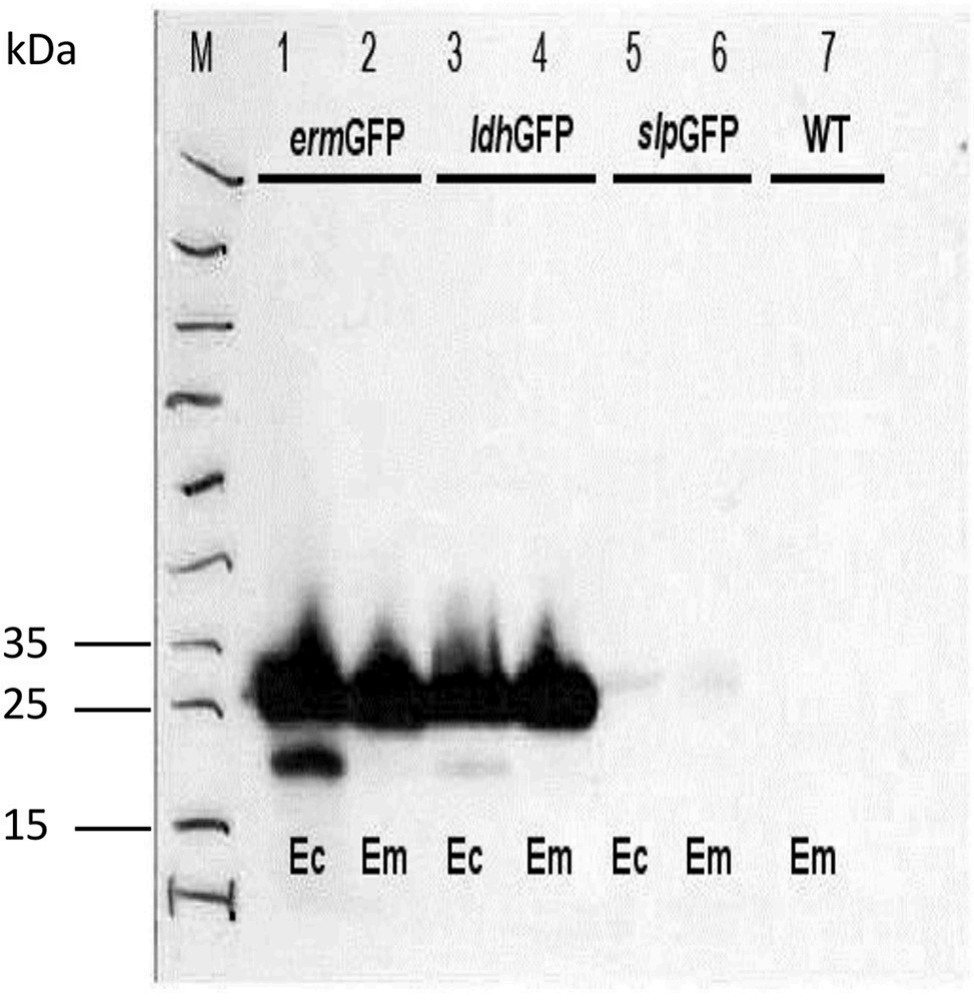
**Comparison of the level of recombinant GFP protein expression in ***E. coli*** DH5α and ***E. mundtii*** strains by western blot**. Bacterial cell lysates from exponentially grown cultures were run through western blot. As immunoblotting gel shows, a significant amount of GFP protein is expressed by *E. coli* and *E. mundtii* strains harboring pTRKH3-*erm*GFP and pTRKH3-*ldh*GFP plasmids, whereas less protein expression can be detected for strains with pTRKH3-*slp*GFP, and no expression is shown for the wild-type strain. Ec, *E. coli*; Em, *E. mundtii*; WT, Wild type, and M, Page Ruler Prestained Protein Ladder. The molecular mass of GFP protein is approximately 27 kDa.

We analyzed the total expressed GFP in *E. coli* DH5α and *E. mundtii*. Western blot results showed that the GFP gene was expressed in large quantities in *E. mundtii* and *E. coli* DH5α cells when the cells were transformed with pTRKH3-*erm*GFP. A thick band associated with the production of large amounts of GFP protein was observed in the immuno-blotting gel for both bacterial cells transformed with pTRKH3-*ldh*GFP-expressing plasmid. Low quantities of protein were produced by the *slp* promoter controlling the GFP expression in both bacteria. The wild-type bacteria did not express GFP protein as expected (Figure 2).

**Figure 3:**
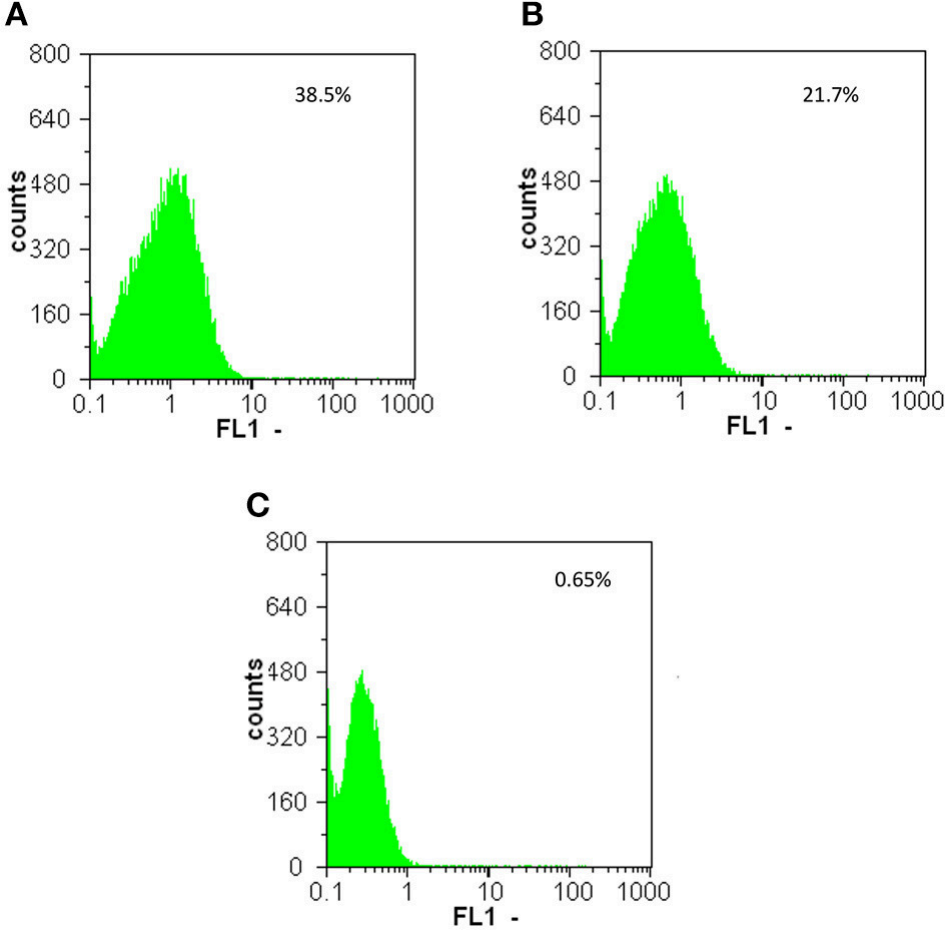
**Flow cytometry histograms of the GFP fluorescent intensities produced by overnight cultures of fluorescent ***E. mundtii*** cells harboring different promoter constructs with (A) pTRKH3/***erm***GFP, (B) pTRKH3/***ldh***GFP, and (C) pTRKH3/***slp***GFP**. The intensities decrease from left to right, with the highest for contructs with *erm* promoter (38.5%), *ldh* (21.7%), and *slp* (0.65%) as the least efficient promoter. y-axis represents the number of bacterial counts and x-axis represents the fluorescence intensity.

Flow cytometry analysis confirmed the results obtained by epifluorescence microscopy and western blot. As expected, overnight cultures of *E. mundtti* cells with pTRKH3-*erm*GFP were highly fluorescent (38.5%), whereas cultures of pTRKH3-*ldh*GFP (21.7%) were slightly fluorescent and those of pTRKH3-*slp*GFP (0.65%) showed almost no fluorescence (Figures 3A–C). Due to the efficiency of pTRKH3-*erm*GFP, this construct was chosen to transform *E. mundtii* and used for feeding experiments.

### Colonization of the intestinal tract of *S. littoralis* with genetically tagged bacteria

**Figure 4:**
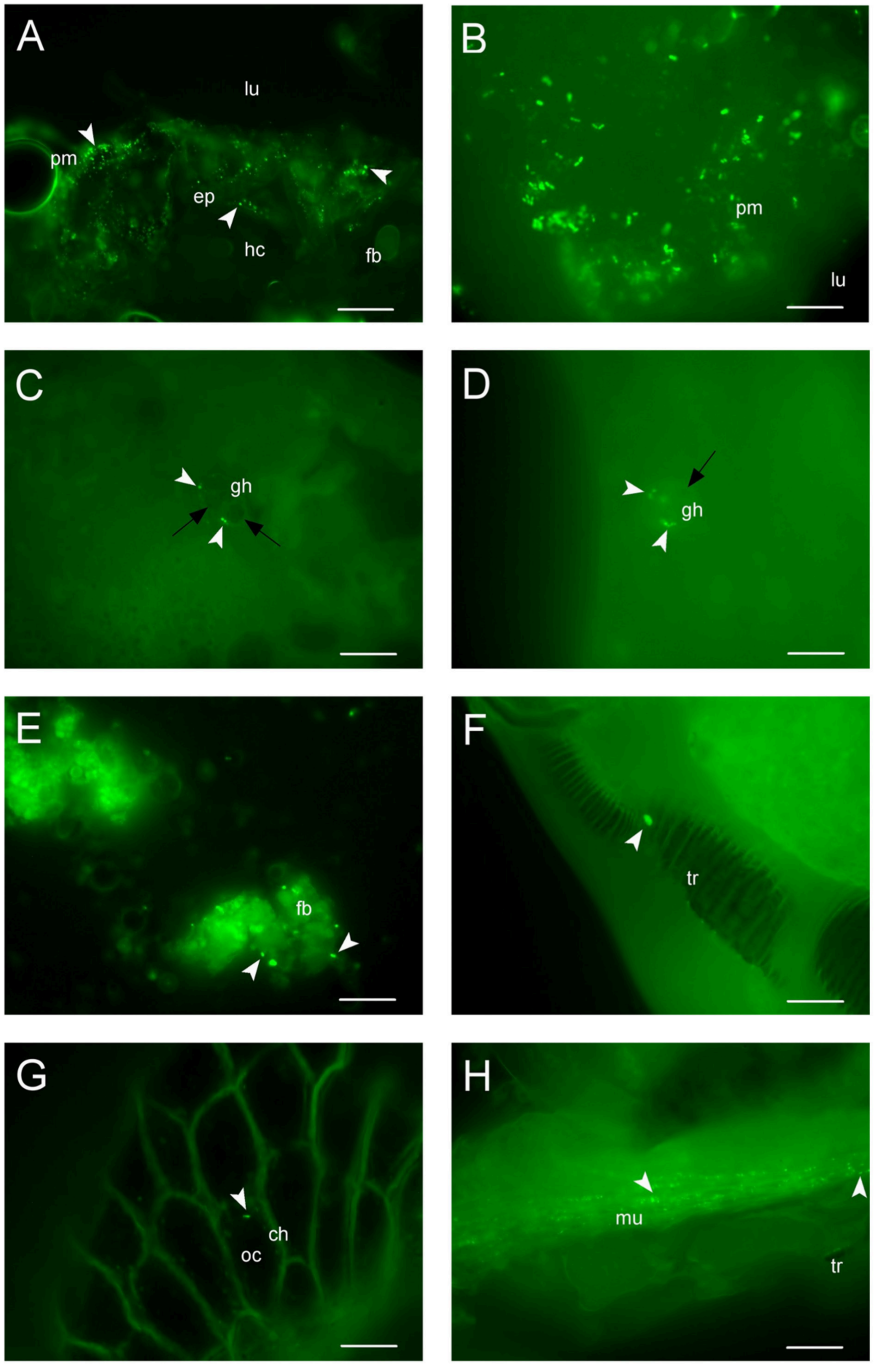
**Colonization of GFP-expressing ***E. mundtii*** in the ***Spodoptera*** intestinal tract. (A)** Fluorescent image of the foregut region of fourth-instar larvae: bacteria are immobilized around the peritrophic membrane and gut epithelium (arrowheads) located adjacent to the hemocoel and fat body, scale bar = 20 μm. **(B)** In fifth-instar larvae, large clumps of bacteria are attached to the peritrophic membrane of the midgut tissue, scale bar = 10 μm. **(C,D)** Histological sections show fluorescent bacteria (arrowheads) are trapped within granular hemocytes containing nodules (black arrows) in the hindgut and midgut, leading to phagocytosis at the end of larval life, the sixth instar, scale bars = 10 μm. **(E)** A few bacteria are attached (arrowheads) to the fat body of pupae showing bacterial lysis occurs. **(F)** A single viable bacterial cell is immobilized in the tracheole of the adult, scale bar = 5 μm. **(G)** The fluorescent *E. mundtii* (arrowhead) is detectable in the oocyte of the eggs, scale bar = 10 μm. **(H)** Clusters of fluorescent bacteria (arrowheads) are scattered in the muscular tissue of the second generation first-instar offspring after hatching, scale bar = 10 μm. ch, chorion; ep, epithelium; fb, fat body; gh, granular hemocyte; hc, hemocoel; lu, gut lumen; mu, musculature; oc, oocyte; pm, peritrophic membrane; tr, tracheole. Magnification, **(A–G)**, 630X; **(H)**, 400X.

GFP-tagged *E. mundtii* were fed to larvae of *S. littoralis* to visually monitor the persistence and fate of the bacteria within the digestive tract of different stages in the life cycle. We observed that fluorescent bacteria multiplied in the foregut, midgut, and hindgut regions after early ingestion of fluorescent bacteria. A high concentration of green fluorescent bacterial cells could be visualized in the foregut and midgut, but decreasing amounts toward the hindgut region of the third-instar larvae (data not shown). During this early stage of ingestion, the density of bacteria was high in most parts of the gut tissues. GFP bacteria were seen scattered in the foregut of the fourth-instar larvae, around the peritrophic membrane as well as entering the epithelium, adjacent to the hemocoel and fat body (Figure 4A).

In the early stages of ingestion the fluorescent bacteria could be seen accumulating in the midgut of fifth-instar larvae (Figure 4B) where they clumped together in the region of the peritrophic membrane. GFP-tagged bacteria were seen starting to double in larvae from fourth to fifth instars (data not shown). In sixth-instar larvae, strikingly, bacteria were trapped in the nodules of granular hemocytes, suggesting the occurrence of phagocytosis (Figures 4C,D). The number of bacteria was reduced significantly in sixth-instar larvae in most parts of the gut. A sharp reduction in the density of fluorescent enterococci occurred during pupation (Figure 4E). Bacteria went from clusters to free-standing groups by attaching to the fat body of pupae; at this stage there was no inner gut in the pupae to keep them from moving around.

Viable fluorescent cells of *E. mundtii* were detected in the tracheole of the adult insect (Figure 4F). This shows bacteria were successfully transmitted from pupal to new adult gut tissue, although there were few or no other bacteria found in the tracheole. We also tested the transmission route of recombinant bacteria by allowing individual adults to mate. Remarkably, we observed that a few bacteria were detected in the oocyte (Knorr et al., 2015) and none in the chorion of the eggs (Figure 4G), which proves GFP-tagged bacteria can survive after almost 30 days at various stages of the entire life cycle of *S. littoralis*. We also showed that, after hatching, fluorescent bacteria were detected in the muscular tissue of the first-instar larvae of second generation offspring (Figure 4H).

### Viable GFP bacterial cell counts

**Figure 5:**
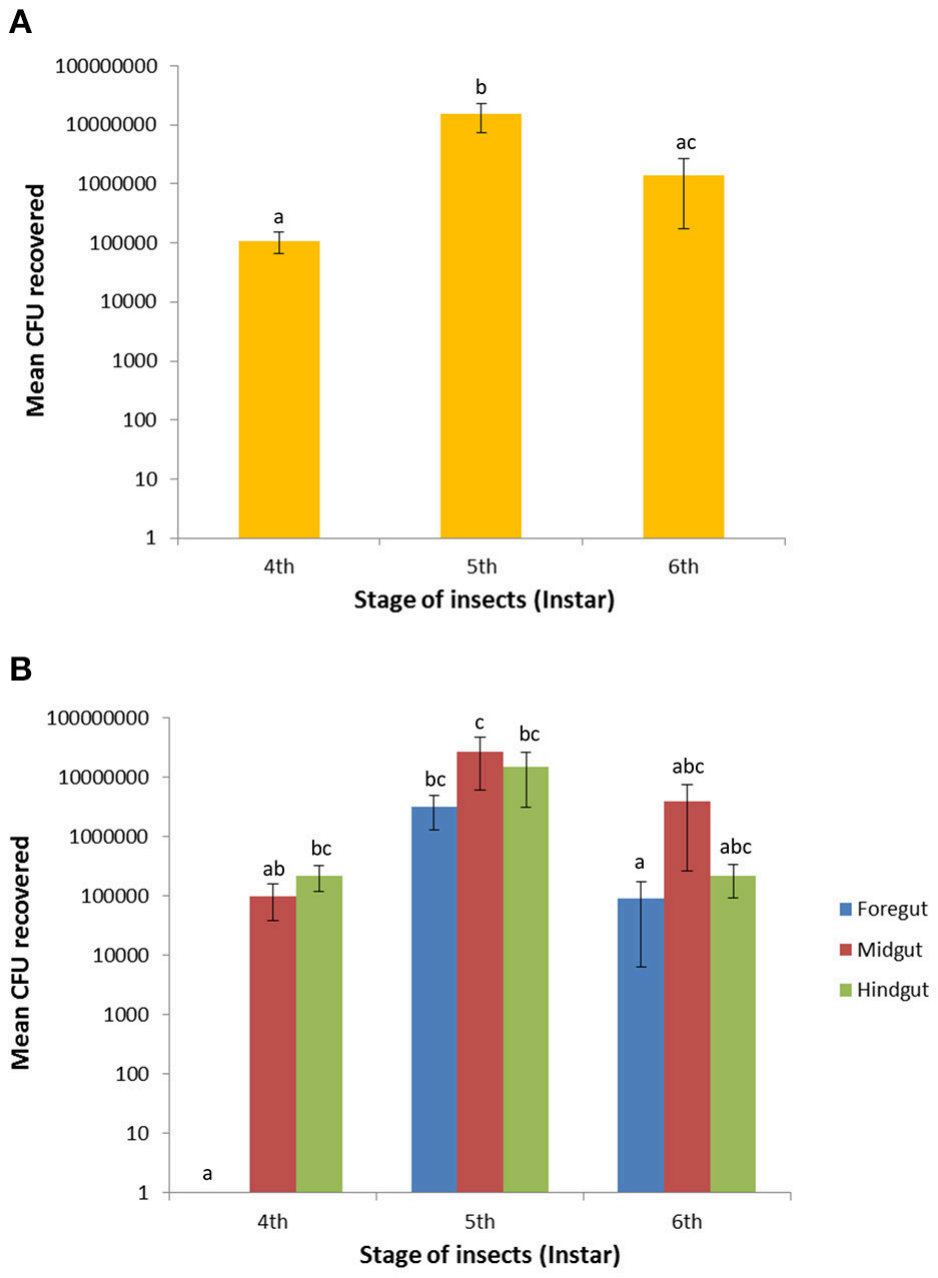
**Recovery of GFP-expressing ***E. mundtii*** in the foregut, midgut and hindgut across larval stages of ***S. littoralis***. (A)** Bacterial recoveries are based on the mean average of the combination of three gut regions of six independent larvae (*n* = 18 per larval stage). **(B)** Bacterial counts were determined in individual gut region within the same digestive tract of each stage (*n* = 6 per gut region). Different letters above error bars (SEM) denote significant differences between pairs (*P* < 0.05). CFUs = colony forming units.

Total fluorescent *E. mundtii* were recovered and counted from individual gut regions (foregut, midgut, and hindgut) on selective THB agar containing erythromycin. The mean of CFUs of bacteria recovered from each gut region showed significant difference between larval stages (*F* = 15.38; *df* = 2; *P* < 0.0001) by one-way ANOVA test. Further pairwise comparison revealed significant differences between the mean number of CFUs of combined gut parts between larvae in the fourth and fifth instars (likelihood ratio: 4.062; *P* < 0.0001) as well as the fifth and sixth instars (likelihood ratio: 3.048; *P* = 0.0006) but not between the fourth and sixth instars (likelihood ratio: 1.014; *P* = 0.3853; Figure 5A).

The number of *E. mundtii* in the foregut region was relatively low until to the fourth instar and transiently raised to 3.2 ± 1.9 × 10^6^ cells (*P* < 0.0001) during the fifth instar, followed by a decrease to 9.2 ± 8.6 × 10^4^ cells (*P* < 0.0053) during the sixth instar. A sharp decrease by 97.1% occurred toward the late-instar larval stage. The midgut CFU count rose during the fourth instar from a mean of 1.0 ± 0.6 × 10^5^ to 2.7 ± 2.1 × 10^7^ at the end of the fifth instar, representing a significant difference (*P* = 0.0425). In the sixth instar, bacterial counts fell to 4.0 ± 3.7 × 10^6^ and showed no significant difference to larvae in the fifth and sixth instars (*P* = 0.1576). Also in the hindgut region there was a transient increase of bacterial counts from 2.2 ± 1.0 × 10^5^ (fourth instar) to 1.5 ± 1.2 × 10^7^ at the end of the fifth instar; this number fell by 94.5% to 2.2 ± 1.3 × 10^5^ at the end of the sixth instar (*P* = 0.1087; Figure 5B). The ± values represent the standard error (SE). For some larvae, the hindgut region did not show any CFUs, possibly due to the high variation in the feeding behavior of individual larva. Overall, the number of bacteria in the intestinal tissues steadily increased from fourth- to fifth-instar larvae, but decreased tremendously during the sixth larval instar. The number of mean CFUs remained low during early pupation and slightly increased in the late pupation and adult stages (data not shown). No CFUs of fluorescent *E. mundtii* were detected from control larvae.

### Tracking of ingested GFP bacteria by colony PCR

**Figure 6:**
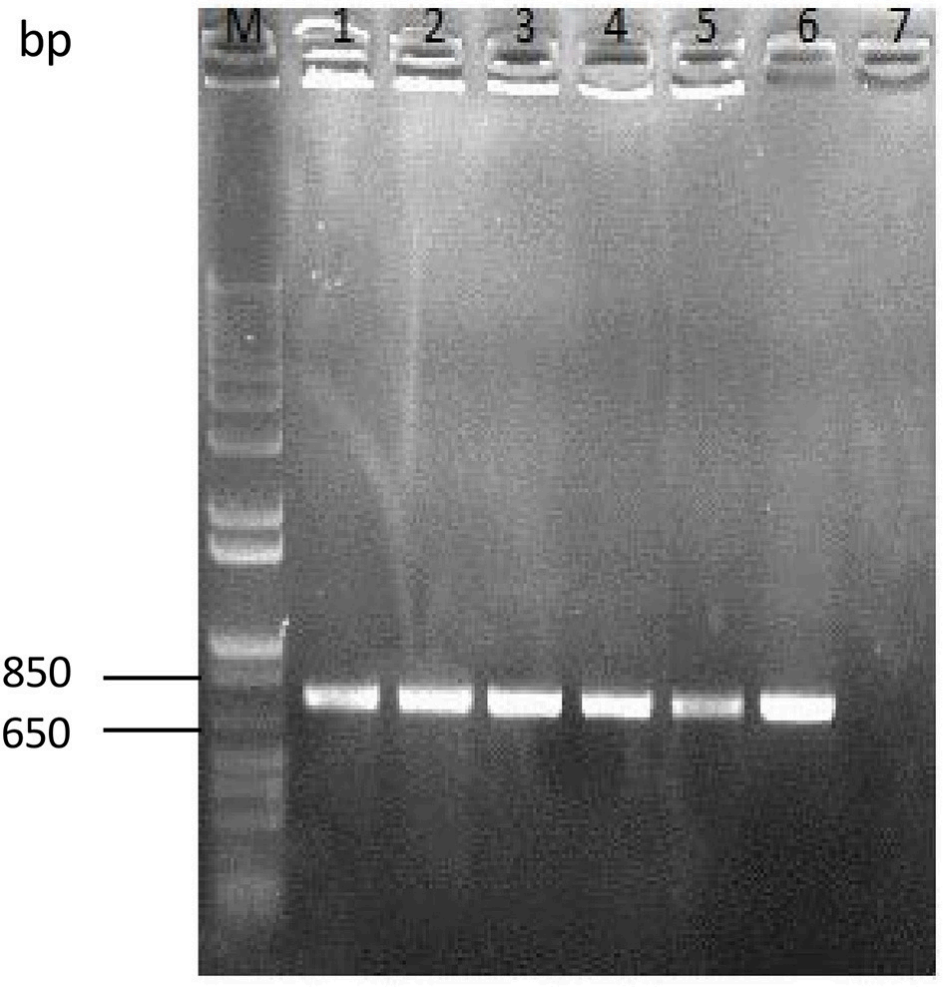
**Colony PCR-amplification of enumerated colonies of ***E. mundtii*** harboring GFP recovered from the intestinal tracts of larvae at different life stages**. The *gfp* gene was amplified from fourth-, fifth,- and sixth-instar pupae and from adult insects. M, molecular weight marker (1-kb Plus DNA ladder, Invitrogen); Lane 1, fourth instar; Lane 2, fifth instar; Lane 3, sixth instar; Lane 4, pupa; Lane 5, adult; Lane 6, positive control (plasmid pTRKH3-*erm*GFP); Lane 7, negative control. The size of *gfp* gene is ~735 bp.

The gut content of different stages of development of the insect was enumerated on selective agar plates. The bacterial colonies grown on agar were picked for colony-PCR experiments to verify the presence of GFP-containing plasmid. We were able to amplify the *gfp* gene of around ~735 bp from bacterial colonies at all stages of development (Figure 6). In addition, we could detect the GFP amplicon in fecal samples of all stages (data not shown), which confirmed that transgenic bacteria were present and could colonize the intestinal tract of *S. littoralis*.

## Discussion

The GFP-expressing plasmids used in this study were derived from a common backbone *E. coli*-enterococcal shuttle vector (pTRKH3), which was controlled by three constitutive promoters of different strengths. This vector contained moderate copy numbers (30–40) in *E. coli* and a high copy number (45–85) in *Streptococcus* and *Lactococcus* species (O'Sullivan and Klaenhammer, 1993; Papagianni et al., 2007). Moreover, it was stably maintained up to 25 generations without erythromycin and lost < 4% after transformation into *Lactococcus lactis* (Papagianni et al., 2007). Our results showed that the strongest GFP expression signal was derived from the *ermB* promoter, which displayed the high fluorescence of recombinant bacteria upon detection by epifluorescence microscopy, western blotting and flow cytometry. This promoter is likely to be highly effective in many *Enterococcus* species, as it is derived from the broad-host range plasmid pAMß1 of *E. faecalis* (Swinfield et al., 1990). In *Staphylococcus aureus*, erythromycin resistance is caused by ribosome methylases encoded by *ermA, ermB*, and *ermC* genes which are involved in the methylation of 23S rRNA (Leclercq, 2002). The addition of erythromycin antibiotic as substrate may increase the expression of *ermB* gene in *E. mundtii*, thus activates the GFP expression. In addition, the pTRKH3 vector which originates from pAMß1 may be suitable for replication in Gram-positive bacteria. The strength of *gfp* gene expression controlled by these promoters was similar to that reported in *Lactobacillus reuteri* strains (Lizier et al., 2010).

The strength of promoter used to drive successful expression of heterologous proteins depends on strain and vary within LAB (McCracken and Timms, 1999). The constitutive *ldh* promoter is highly efficient in *Lactobacillus casei* (Pouwels et al., 2001) as well as in *E. mundtii*. It has been shown that the *ldh* gene is highly active in the logarithmic phase, but its expression decreases in the stationary phase in *Lactobacillus helveticus* (Savijoki and Palva, 1997). The low GFP expression signal from the *slp* promoter in our study may be due to different rate of transcription and translation of S-protein genes. In two *Lactobacillus* species, similar genes can be expressed with different regulatory mechanism (Pouwels et al., 1998). In some species of bacteria, the S-protein genes are controlled by multiple promoters (Vidgren et al., 1992), and some are preceded by a single promoter. The yield of mRNA controlled by multiple promoters might be higher than the yield directed by a single promoter. The regulation of S-protein gene expression is still not very well-known and may be growth-dependent. One of the five promoters upstream of S-protein gene in *Brevibacillus brevis* is active during all growth phases, while another promoter is only active during exponential growth (Adachi et al., 1989). It has been shown that the half-life of the S-protein mRNAs is different between bacterial species, *Aeromonas salmonicida* (22 min; Chu et al., 1993), *Caulobacter crescentus* (10–15 min; Fisher et al., 1988), and *L. acidophilus* (15 min; Boot et al., 1996). Another possible explanation of low GFP expression directed by a single *slp* promoter might be that *E. mundtii* do not synthesize S-layer protein which was also reported in *L. casei* as well (Masuda and Kawata, 1983).

Expression of *gfp* has effect on the physiology and fitness of the bacteria (Rang et al., 2003; Allison and Sattenstall, 2007). It was reported that the growth of *Salmonella* was suppressed due to constitutive expression of *gfp* (Oscar, 2003). In contrast, two case studies using *E. coli* and other pathogenic bacteria showed that the *gfp* expression did not affect bacterial survival (Leff and Leff, 1996; Valdivia et al., 1996). The use of erythromycin as an antibiotic selective marker has a number of drawbacks. It may cause toxic effects on the host insect and other gut microbes. The excessive use of antibiotics causes its spread in the environment and thus produces many antibiotic resistant pathogenic bacteria (Hamer and Gill, 2002; Livermore, 2007; Walsh and Fanning, 2008). Under laboratory conditions, it has been shown that the antibiotic resistance genes via a plasmid can be transferred into foodborne pathogenic bacteria by turning antibiotic sensitive strains into resistant ones (Van Meervenne et al., 2012).

Researchers have found that indigenous bacteria derived from the host insect could be reintroduced and could survive in the native gut environment (Chapco and Kelln, 1994; Dillon and Charnley, 1996; Martinez-Sanudo et al., 2011). In previous experiments, we introduced GFP-tagged *E. coli* into the gut of *S. littoralis* and were able to monitor the bacteria for up to 4 days after which they disappeared (Wallstein, 2014). Our observation was independently confirmed by others (Thimm et al., 1998), who found that genetically modified non-indigenous *E. coli* vanished within 1 day after introduction into the gut of collembola. In contrast, the indigenous *Alcaligenes faecalis* was able to colonize the intestinal tract of *Folsomia candida* (Collembola) for about 2 months (Thimm et al., 1998). In other studies, Husseneder and Grace failed to produce a persistent population of transgenic *E. coli* in the guts of termites (Husseneder and Grace, 2005). However, they successfully introduced the genetically modified indigenous *Enterobacter cloacae* in termite guts, where the bacteria persisted for almost 3 months. The failure of non-indigenous *E. coli* to establish a stable population in the guts of termites may be due to resistance by the indigenous gut bacteria (Dillon and Dillon, 2004), and the fact that the non-indigenous bacteria might be outclassed by the natural microbial flora (Chao and Feng, 1990; Leff and Leff, 1996).

Since it is of interest to study the mode of transmission of GFP-labeled *E. mundtii* to the next generation, we also analyzed the occurrence of GFP-labeled cells in pupae, and oocytes of *S. littoralis*. The fluorescent bacteria were found, indeed, in the oocytes and were transmitted to the second-generation larvae. Recent examinations using fluorescent bacteria have found the bacteria to be transmitted from the gut into the eggs in *T. castaneum* (Knorr et al., 2015). In one hypothesis, the egg-smearing mode of vertical transmission, the surface of the eggs is contaminated with the environmental bacterial symbionts, which the freshly hatched larvae acquire by feeding on the eggshell (Douglas and Beard, 1997; de Vries et al., 2001). Bakula showed that the methylene blue dye used to stain the embryos of *Drosophila* was detected in the intestine of hatched first-instar larvae, suggesting that the larvae had ingested the embryos (Bakula, 1969). Transmission through parents also occurs, either from the mother to the offspring or from the father to mother and then to the offspring (Moran and Dunbar, 2006; Damiani et al., 2008). Damiani et al. also demonstrated that male-borne symbionts of the bacteria of the genus *Asaia* were transferred to females during mating of *Anopheles stephensi* mosquitoes (Damiani et al., 2008). These bacteria were then further transmitted from the mother to the offspring during sexual reproduction. In separate studies, Moran and Dunbar also showed that it is possible, though rare, for symbionts to be transferred from the father to the offspring (paternal transfer) in aphids (Moran and Dunbar, 2006).

Several factors—for instance, pH and oxygen availability can shape microbial colonization in different gut niches. It is known that the pH inside the gut of Lepidoptera such as *S. littoralis* is highly alkaline (pH ~ 8.5–10) in the foregut and midgut, and neutral (pH 7.0) in the hindgut (Funke et al., 2008). It has been reported that there is relatively low diversity of bacteria in the extremely alkaline guts of gypsy moth larvae, *Lymantria dispar* (Broderick et al., 2004) and high bacterial density in the low-alkaline guts of larvae in beetles (Coleoptera), flies (Diptera), or bees (Apoidea) (Kadavy et al., 1999; Egert et al., 2003; Mohr and Tebbe, 2006). The survival of *E. mundtii* in alkaline environment shows that it has developed adaptation mechanisms.

It has been shown through FISH analyses that enterococci can form a biofilm-like structure by attaching themselves to the mucus layer of the gut epithelium (Koch and Schmid-Hempel, 2011; Engel et al., 2012; Shao et al., 2014). In our study, interestingly, most of the GFP-tagged bacteria did not spread throughout the whole gut content but were confined within the mucus layer of the peritrophic membrane. This membrane prevents the bacteria from gut lumen from entering the epithelium, as reported in the study of *Bactrocera oleae* (Mazzon et al., 2008). The peritrophic membrane was shown to have a defensive role against pathogens in *Drosophila melanogaster* (Kuraishi et al., 2011) and to act as a barrier against food particles and digestive enzymes (Lehane, 1997; Hegedus et al., 2009). In addition, the membrane was able to protect the bacteria from unfavorable gut conditions such as alkaline and acidic pH (Crotti et al., 2009). In our case, we observed that the fluorescent bacteria crossed the peritrophic membrane and invaded the gut epithelium of the fourth-instar larvae.

The composition and density of microorganisms changed as insects aged, for example in the case of the fruit fly, *D. melanogaster* (Ren et al., 2007; Buchon et al., 2009; Storelli et al., 2011; Wong et al., 2011). In our study, the number of fluorescent bacteria increased throughout the larval stage, from fourth- to fifth-instar larvae. This number was significantly higher in tissues from the midgut than from those in the foregut and hindgut, supporting the hypothesis that the midgut is a crucial region for digestion. Beneficial bacteria may be needed to aid in the metabolic activity of host insect. A strong decline of recombinant bacteria was observed in the sixth-instar larvae. This reduction prior to the pupal stage may be associated with the enhanced expression of antimicrobial peptide genes which has been shown in a few previous studies (Samakovlis et al., 1990; Tryselius et al., 1992; Tzou et al., 2000).

Humoral responses, such as the production of antimicrobial peptides, reactive oxygen species, and lysozymes, as well as activation of the prophenoloxidase system, are noticed when microorganisms invade (Jiang, 2008; Tsakas, 2010). Antimicrobial peptides can be repressed by transcription factors, including the homeobox gene *caudal*, in order to retain beneficial gut bacteria in the host insect (Ryu et al., 2008). Remarkably, we detected the encapsulation of fluorescent *E. mundtii* within the nodules of granule hemocytes which leads to bacterial lysis. The two most abundant hemocytes present in the larvae of Lepidoptera are granular cells (granulocytes) and plasmatocytes (Ratcliffe, 1993; Strand and Pech, 1995). The processes of phagocytosis, nodulation and encapsulation are hemocyte-mediated immune responses (Strand, 2008; Tsakas, 2010). Hemocytes encapsulate various cells ranging from bacteria to yeast and even synthetic beads and particles of India ink (Yokoo et al., 1995; Hernandez et al., 1999; Da Silva et al., 2000). We observed that the fluorescent enterococci survived pupation and became transmitted to the adults. Accordingly, the bacteria can be successfully transmitted during metamorphosis escaping the removal or reduction of midgut bacteria by colonizing sites farther away from the meconium. Another explanation may be that bacteria are resistant to the antimicrobial exuvial fluids that are consumed as part of the ecdysial process (Moll et al., 2001).

## Conclusion

We have succeeded in tagging *E. mundtii* (strain KD251) with the *gfp* gene. The recombinant strain that harbors the pTRKH3-*erm*GFP plasmid was chosen to be reintroduced into *S. littoralis*. Interestingly, the fluorescent bacterial cells were able to colonize the intestinal tract of the host insect for nearly 30 days. These bacteria were efficiently transmitted from larval stages to the adult stage, where they survived up to the second generation. Increased knowledge of the distribution and transmission route of indigenous gut symbionts may lead us to better understand their biological role in the host insect.

## Author contributions

Conceived and designed the experiments: BT, YS, WB. Performed the experiments: BT, JA. Analyzed the data: BT. Wrote the paper: BT and WB.

### Conflict of interest statement

The authors declare that the research was conducted in the absence of any commercial or financial relationships that could be construed as a potential conflict of interest.

## Footnotes

^1^JMP® Version 12.1.0. SAS Institute Inc., Cary, NC, USA, 2015.
